# Post-ostial kink of the uterine artery—a technical hurdle in the UAE learning curve

**DOI:** 10.1186/s42155-026-00687-7

**Published:** 2026-04-09

**Authors:** Warren Clements

**Affiliations:** 1 Department of Radiology, Bayside Health, Alfred Care Group, 55 Commercial Road, Melbourne, VIC 3004 Australia; 2 Department of Surgery, School of Translational Medicine, Monash University, Melbourne, Australia; 3 National Trauma Research Institute, Melbourne, Australia

**Keywords:** Embolisation, Fibroid, Uterine artery, Catheter, Stenosis

## Abstract

This technical note describes the “post-ostial kink” which is a phenomenon not known to be previously described, of apparent stenosis approximately 1–2 cm from the uterine artery ostium. This can cause technical difficulties in some patients during uterine artery embolisation (UAE).

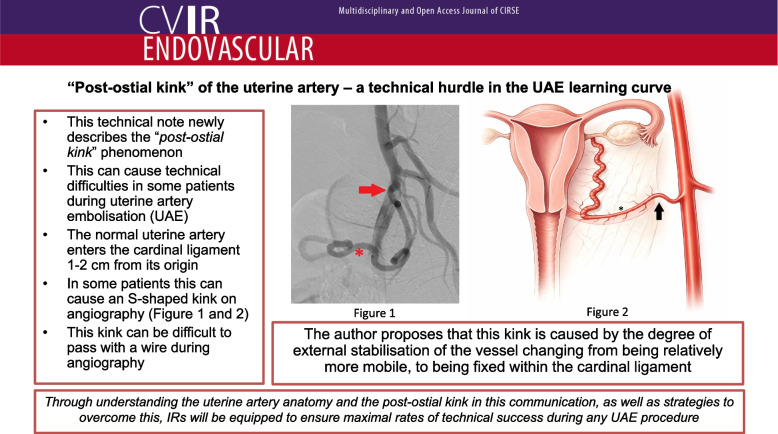

## The normal uterine artery course

The uterine artery commonly arises as one of the first branches of the anterior division of the internal iliac artery [1], although there are many described anatomic variations [2, 3]. From its retroperitoneal location, it courses medially through the cardinal and broad ligaments, giving off branches to the fallopian tube and ureter. The artery bifurcates into ascending and descending branches near the uterus, with the descending branch supplying blood to the cervix and upper vagina [2]. The tortuous ascending branch supplies blood to the uterine body and fundus. Positioned medial to the ascending branches are arcuate arteries which penetrate the serosa of the uterus and enter the myometrium, continuing inward as the radial arteries [4]. These radial arteries enter the endometrium and give rise to the spiral arteries. Radial and spiral arteries are typically small, less than 300 µm in calibre [5].

### Finding and navigating the uterine artery

**Fig. 1:**
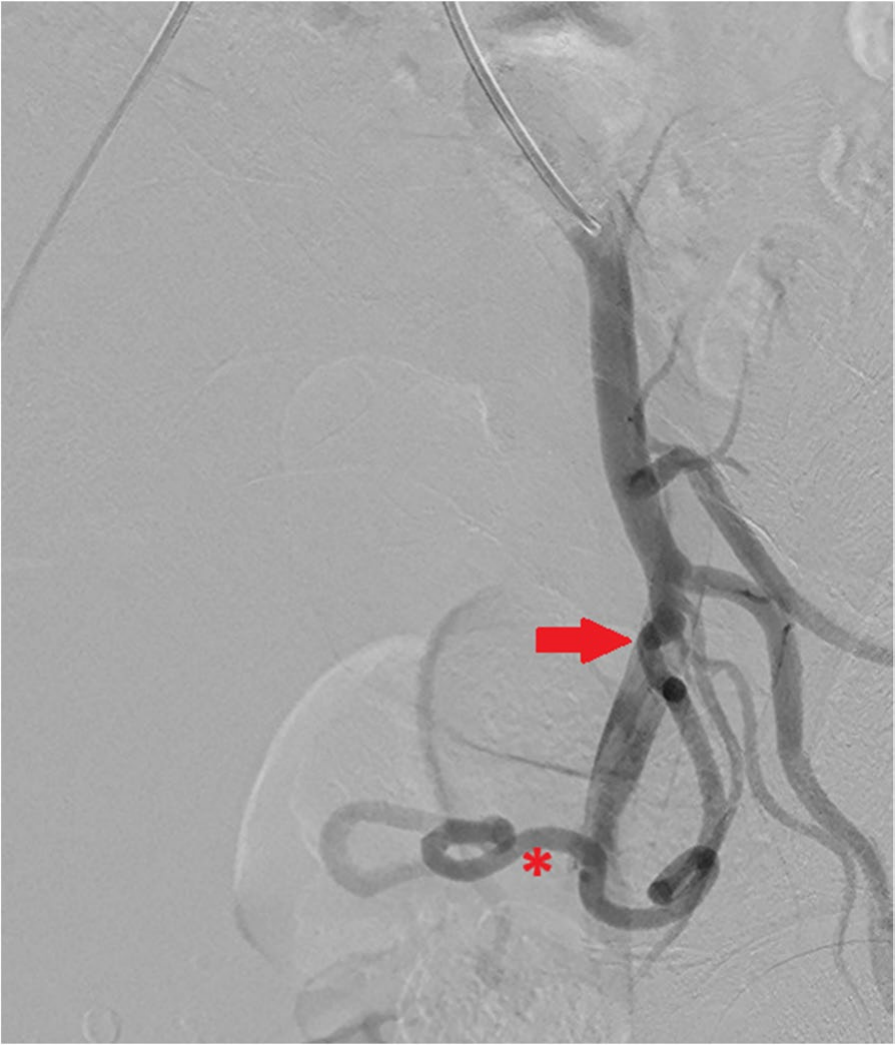
Left internal iliac artery angiogram with 27 degrees of ipsilateral oblique. The uterine artery (asterisk) is dilated and shows a characteristic tortuous appearance. There is an s-shaped kink near the ostium of the uterine artery (solid arrow)

**Fig. 2:**
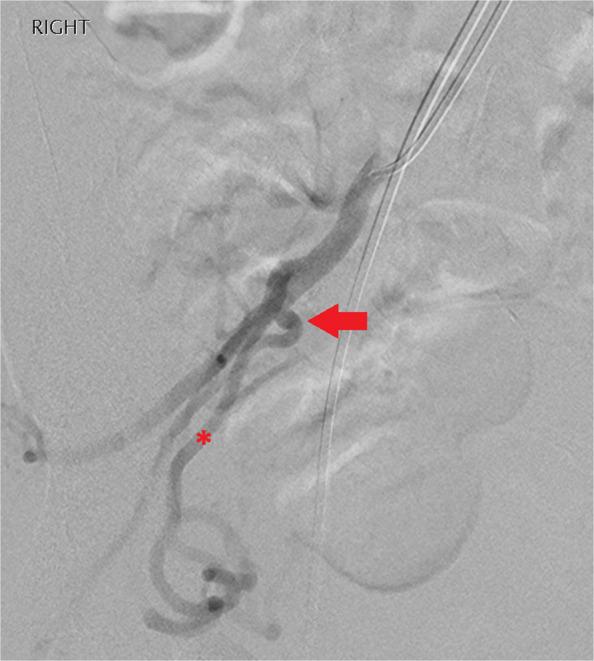
Right internal iliac artery angiogram with 35 degrees of ipsilateral oblique, using a Waltman-loop to access. The uterine artery (asterisk) is dilated. There is an s-shaped kink near the ostium (solid arrow). Note this is a different patient to Fig. 1

Angiographically, the uterine artery is identified with an ipsilateral oblique projection of approximately 25–40 degrees [6]. Using iodinated contrast between 50 and 100% strength, angiography will usually project the uterine artery as the first visible medial branch with its characteristic tortuous appearance (Fig. 1). The author proposes that an angiographic observation seen in some women is that the artery may make an “S” shape approximately 1–2 cm after its origin, newly termed the post-ostial kink (Fig. 2). This kink is characteristically not associated with delay or inhibition to flow, is not the result of vasospasm, and does not behave like the remaining tortuous uterine artery which can be easily navigated.

### What is the post-ostial kink?

As the uterine artery passes medially, it enters the cardinal ligament [7]. The cardinal ligament is a focal thickening at the base of the broad ligament and has a structural role in stabilising the uterus and vagina. The uterine artery passes through the cranial portion of the ligament in a space between two bands [8].

**Fig. 3:**
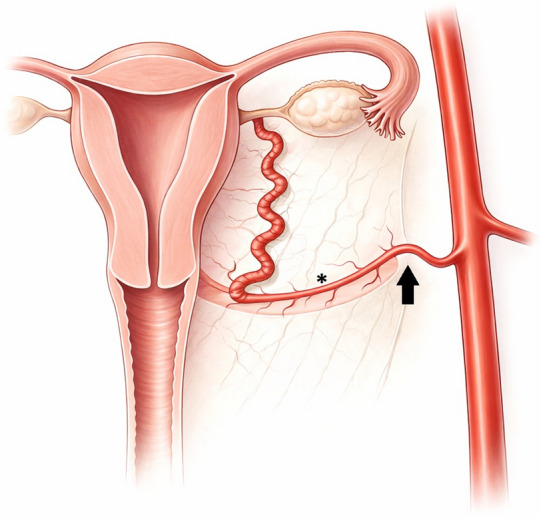
Illustration showing the course of the uterine artery (asterisk) relative to the broad ligament. The intersection of these structures (solid arrow) is anatomically co-localised with the post-ostial kink described in this letter. Image created with the use of ChatGPT-5.2

The author theorises that based on anatomic correlation and the authors’ experience, the post-ostial kink phenomenon occurs at the point of uterine artery insertion into the cardinal ligament. Here, the degree of external stabilisation of the vessel changes from being relatively more mobile to being fixed within the ligament (Fig. 3). This may mean that the angle of insertion and/or change in external vessel stability may cause difficulties passing a wire past this point, even if the kink does not appear haemodynamically stenotic on angiography.

### How to overcome the post-ostial kink during UAE: soft or firm?

The default approach in the author’s experience is to use a 5-French 0.035″ base catheter positioned in the internal iliac artery to obtain adequate arterial mapping. After placing an image overlay, an 0.018″ high-flow microcatheter (Progreat 2.7 Fr, Terumo, Japan) can be advanced through the base catheter until the desired embolic location is reached. The use of a microcatheter is favoured by the author as it ensures uterine artery flow is maintained. When the post-ostial kink is significant, the author’s experience is that the microwire will enter the ostium of the uterine artery for approximately 1–2 cm but will not pass beyond the kink easily. Some initial strategies to overcome this may be to re-shape the wire using an exaggerated angle or by making an s-shape with the wire tip.

Based on the authors’ experience, if basic steps are unsuccessful, the first troubleshooting option is to use a “soft” approach which involves using a softer or more narrow calibre wire — for example, 0.016″ Synchro Select, Stryker, Michigan, Unites States. This allows the wire to pass through the kink, and when sufficient wire is introduced, the base catheter can follow. The author observes that in this setting, the uterine artery often straightens after the wire is passed, confirming the kink as being dynamic and anatomic, not a pathology such as vasospasm.

If this approach is not successful, the second option is to take a “firm” approach. This involves using the 0.035″ base catheter and an atraumatic angled 0.035″ wire (Radiofocus Glidewire, Terumo, Japan). This wire is less malleable but more likely to straighten the kink, allowing passage of the base catheter into the uterine artery. The “firm” approach is best performed with a relatively softer base catheter (Glidecath, Terumo, Japan) and/or using a smaller calibre 4-French catheter. Once the base catheter passes the kink, it may be used for embolic delivery; however, flow dynamics should be assessed first as there is a risk of ostial occlusion and/or vasospasm. Alternatively, the wire may be exchanged for an 0.018″ microcatheter, and once the microcatheter is positioned in the desired embolic location, the base catheter can be withdrawn under fluoroscopic guidance until it has been repositioned back in the internal iliac artery, as this often improves uterine artery flow. The “firm” approach may be challenging with smaller uterine arteries (e.g. non-dominant side, small fibroids, or when treating adenomyosis) or for the ipsilateral side if using certain techniques (e.g. Waltman-loop technique or the Roberts catheter). As such, the author’s approach and teaching to trainees is to generally use the “soft” technique first when feasible.

In conclusion, the post-ostial kink is a phenomenon of apparent stenosis approximately 1–2 cm from the uterine artery ostium, and anatomically the author theorises that this corresponds with the site of arterial insertion into the cardinal ligament near the pelvic brim. It is dynamic and not clinically significant for the uterus but often results in technical challenges for the IR when performing UAE. It is essential to understand this phenomenon and that troubleshooting tools are available to ensure UAE can be performed bilaterally and thus achieve technical success approaching 100% in line with standards of practice documents [6, 9].
